# Esculetin Combats Multidrug-Resistant *Salmonella* Infection and Ameliorates Intestinal Dysfunction via the Nrf2 Pathway

**DOI:** 10.3390/antiox13101170

**Published:** 2024-09-26

**Authors:** Wenjiao Xu, Wenjun Ding, Liyan Jia, Kui Zhu, Qingfeng Luo

**Affiliations:** 1 Department of Gastroenterology, Beijing Hospital, National Center of Gerontology, Institute of Geriatric Medicine, Chinese Academy of Medical Science, Beijing 100730, China; xwjvet@163.com; 2 College of Life Sciences, University of Chinese Academy of Sciences, Beijing 100049, China; dingwj@ucas.ac.cn; 3 National Key Laboratory of Veterinary Public Health and Safety, College of Veterinary Medicine, China Agricultural University, Beijing 100193, China; 18701361624@163.com

**Keywords:** coumarin, esculetin, intestinal barrier, multidrug-resistant (MDR), Nrf2, *S*. Tm

## Abstract

The increasing incidence of multidrug-resistant (MDR) *Salmonella enterica* serovar Typhimurium (*S*. Tm), known for causing invasive enteric infections, presents a significant public health challenge. Given the diminishing efficacy of existing antibiotics, it is imperative to explore novel alternatives for the treatment of MDR *S*. Tm infections. Here, we identified esculetin (EST), a natural coumarin abundant in dietary foods and herbs, as a compound exhibiting broad-spectrum antibacterial properties against a range of MDR bacteria. Our findings demonstrate that EST effectively inhibited the proliferation and expansion of MDR *S*. Tm in both in vitro experiments and animal models. Specifically, EST significantly downregulated the type 3 secretion system-1 (T3SS-1) virulence expression of MDR *S*. Tm, thereby preventing its invasion into intestinal epithelial cells. In *S*. Tm-infected mice, we observed cecal injury characterized by the upregulation of inflammatory cytokines, a reduction in goblet cell numbers, a decreased expression of tight junction proteins, and microbial dysbiosis. Conversely, EST treatment ameliorated these pathological changes induced by *S*. Tm infection and reduced oxidative stress by activating the nuclear factor erythroid 2-related factor 2 (Nrf2) signaling pathway, thereby improving intestinal barrier function. These results suggest that dietary coumarins or a targeted plant-based diet may offer a promising strategy to counteract MDR bacteria-induced enteric diseases.

## 1. Introduction

*Salmonella enterica* serotype Typhimurium (*S*. Tm), an important enteropathogenic bacterium, is among the most common agents of invasive *Salmonella* infections [1]. These infections range in severity from self-limiting gastroenteritis to invasive typhoid fever [2], raising substantial public health concerns worldwide [3]. Annually, *S*. Tm is responsible for approximately 535,000 infections and 80,000 deaths [4]. For instance, in 2022, an outbreak of *S*. Tm linked to contaminated food products affected at least 113 countries [5]. To proliferate extensively in the gut, *S*. Tm employs multiple virulence genes situated within *Salmonella* pathogenicity islands (SPIs). Among these, SPI-1 encodes type 3 secretion system-1 (T3SS-1), which facilitates the transfer of secretory effectors into host cells, assisting *S*. Tm to invade the intestinal epithelium and triggering acute intestinal inflammation and diarrhea [6].

Although antibiotics remain the primary therapy for bacterial infections, the efficacy of conventional antibiotic regimens is increasingly compromised by the rise in multidrug-resistant (MDR) *S*. Tm strains. Notably, colistin, a last-resort antibiotic against MDR Gram-negative pathogens [7], is becoming less effective due to the emergence of MDR *S*. Tm strains carried with mobilized colistin resistance genes (*mcr*). These resistant strains have been identified in humans, animals, and environmental samples across 12 countries on all continents, except Antarctica and Africa [8]. Consequently, there is an urgent imperative to investigate effective alternatives and strategies to combat MDR *S*. Tm infections.

The development and spread of drug resistance are outpacing the introduction of new antibiotics into clinical use. Compared to synthetic chemotherapeutic drugs, antibacterial natural products offer a promising approach to combating MDR bacteria [9]. Among a variety of natural products, coumarins have garnered intense attention due to their broad pharmacological activities, including antibacterial, anticancer, anti-inflammatory, antioxidant, and neuroprotective effects [10,11]. Over 1300 natural coumarins have been isolated from 30 plant families, such as *Apiaceae*, *Clusiaceae*, *Caprifoliaceae*, *Guttiferae*, *Nyctaginaceae*, *Oleaceae* and *Umbelliferae* [12]. Daily exposure to coumarin for individuals is notably substantial [13], with coumarin derivatives widely distributed in diverse foods and herbs such as tea, wine, cocoa, apples, grapes, and cloudberry [14,15,16]. Among these, esculetin (EST) and its glycoside esculin are active ingredients of the traditional Chinese medicine Cortex Fraxini. Compared to esculin, EST has been reported to possess robust antibacterial activities against various pathogenic bacteria [17]. Additionally, EST is a natural antioxidant with significant antioxidant and anti-inflammatory properties, which are crucial since oxidative stress plays a key role in the pathogenesis of diverse diseases, including bacterial infections [18]. However, there have been few studies examining whether EST alleviates oxidative stress and intestinal barrier dysfunction, thereby inhibiting MDR *S*. Tm-mediated enteric infections.

To address this, we first conducted a series of in vitro experiments to investigate the impact of EST on the growth and cell invasion of MDR *S*. Tm. Subsequently, we established animal models to evaluate the therapeutic effects of EST on the proliferation of MDR *S*. Tm and the associated disease severity. Additionally, we explored the effects and mechanisms of EST on oxidative stress levels and intestinal barrier function in *S*. Tm-infected mice. These findings can offer novel insights into potential therapeutic alternatives for combating MDR enteric infections.

## 2. Materials and Methods

### 2.1. MIC Determination by Broth Microdilution

**Figure 1:**
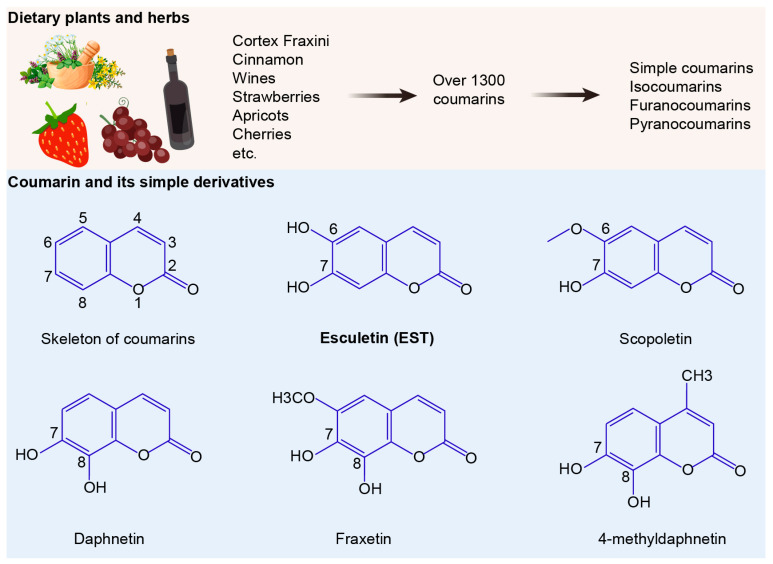
Sources and structures of coumarins. The numbers 1 to 8 indicate the positions of carbon atoms in the chemical structure.

Structurally, coumarins share a benzopyrone skeleton, comprising a planar aromatic ring fused with a lactone moiety. These compounds can be categorized into four groups: simple coumarins, isocoumarins, furanocoumarins, and pyranocoumarins (Figure 1). To evaluate the antibacterial activities of coumarin derivatives, we selected six coumarins and assessed their efficacy using the broth microdilution method, following the CLSI 2021 guidelines. The compounds tested included coumarin, EST, fraxetin, daphnetin, scopoletin and 4-methyldaphnetin (Sigma-Aldrich, St. Louis, MO, USA). Each compound underwent a two-fold serial dilution in Mueller Hinton Broth (MHB) and was then mixed with bacterial suspensions. Incubation at 37 °C for 16–18 h followed. MIC values were determined as the lowest concentrations of each compound that effectively suppressed visible bacterial growth.

### 2.2. Bacterial Growth Curve

Bacteria were inoculated into 1 mL of brain heart infusion (BHI) broth and incubated at 37 °C for 16 h with agitation at 200 revolutions per minute (r.p.m). The bacterial cultures were then diluted 1:100 and standardized to approximately 1 × 10^6^ colony-forming units per milliliter (CFU/mL) in a 96-well microplate. Next, EST was added to the bacterial dilutions, resulting in final EST concentrations of 0, 0.32, 0.64, and 1.28 mg/mL, respectively. Bacterial growth curves were measured by recording at optical density at 600 nm (OD600) at 1 h intervals using the Infinite M200 Microplate Reader (Tecan, Männedorf, Switzerland).

### 2.3. S. Tm Invasion Assay

Intestinal epithelial cells (IEC-6) were seeded at a density of 1 × 10^5^ cells/mL into 12-well plates. The plates were then placed in a 37 °C incubator with 5% CO_2_ for a duration of 12 h. For the *S*. Tm group, *S*. Tm (1 × 10^6^ CFU/mL) was introduced into each well and subsequently incubated for 6 h. In the EST-treated group, *S*. Tm (1 × 10^6^ CFU/mL) and EST were simultaneously added to each well and incubated for 6 h. Following incubation, the IEC-6 cells were washed with phosphate-buffered solution (PBS) and treated with gentamicin (200 μg/mL for 30 min) to eliminate non-invaded *S*. Tm.

For intracellular bacterial count, after an additional washing step, IECs were lysed with 1 mL of 0.5% Triton X-100 for 10 min at 37 °C to release the invaded *S*. Tm. Subsequently, a 100 μL aliquot from each invasion experiment was serially diluted in PBS and plated on TSA agar. Colony counting was conducted following 18 h of incubation at 37 °C.

### 2.4. Confocal Immunofluorescence Microscopy

To visualize *S*. Tm infection, IECs were uniformly spread on glass slides and subsequently infected with *S*. Tm according to the method described above. Following infection, cells were fixed with 4% paraformaldehyde for 15 min and then permeabilized with 0.5% Triton X-100 for 10 min. The cells were then incubated with a mouse anti-*Salmonella* Typhimurium LPS antibody (Abcam, Cambridge, UK, ab8274) for 1 h, followed by incubation with a goat anti-mouse IgG secondary antibody for 1 h. Subsequently, the samples were stained with rhodamine phalloidin (red) and DAPI (blue) for 20 min and 2 min, respectively. Cellular samples that had been fixed and stained were captured as static images using a Leica SP8 confocal microscope (Leica Microsystems, Wetzlar, Germany). Three-dimensional (3D) images were generated by scanning all sections along the X, Y, and Z axes. To determine the location of internalized bacteria, sections along the Z-axis were cut at intervals of 1 or 2 µm. The images were then analyzed and merged using LAS X Core 3.7.4 software (Leica, Wetzlar, Germany).

### 2.5. Quantitative Reverse Transcription-PCR (RT-qPCR) Analysis

Protocols were followed as previously reported [19]. Briefly, RNA was extracted from *S*. Tm 15E475 strains from the respective groups. RNA reverse transcription was carried out using 1 μg of RNA to synthesize cDNA, following the manufacturer’s instructions for the Evo M-MLV Mix Kit (Accurate Biology, Changsha, China). RT-PCR amplification was conducted using the ABI QuantStudio 7 detection system (Applied Biosystems, Waltham, MA, USA). The expression levels of virulence genes were standardized relative to the housekeeping gene *rpoD* in *S*. Tm. Details of the primers utilized are provided in Table 1.

### 2.6. Intestinal Infection of S. Tm

Female C57BL/6 mice, aged 8–10 weeks and weighing 18–20 g, were sourced from SPF (Beijing) Biotechnology Co., Ltd. (Beijing, China) The mice were acclimated for a week under standardized conditions before performing experiments. The mouse model infected with *S*. Tm was established following established protocols described in previous publications [21]. In brief, mice were pre-treated with streptomycin (Antibiotic, Abx) administered as a 100 μL solution of 200 mg/mL in sterile PBS, 24 h before colonization with 10^7^ colony-forming units (CFUs) of *S*. Tm. Feces were collected at specified time points to determine the *S*. Tm load. In the EST-treated group, mice were administered EST (100 mg/kg) 24 h post *S*. Tm-infection as described in a previous study [21]. Mice were euthanized via cervical dislocation 96 h post infection. Intestinal segments, livers, and spleens were collected for subsequent analysis. Feces, intestinal contents, and tissue samples were weighed, homogenized, and cultured on *Salmonella* chromogenic agar plates containing 50 μg/mL ampicillin to determine the CFU per gram of tissue.

### 2.7. Histological Analysis

For the histological examination, liver, spleen, and intestinal tissues were promptly immersed in 4% paraformaldehyde and fixed for 48 h. Subsequently, they were embedded in paraffin and sectioned into 5 μm thick slices. The tissue sections were subjected to staining with periodic acid–Schiff (PAS) or hematoxylin and eosin (H&E). Images of each stained sample were captured using an Olympus microscope.

### 2.8. Enzyme-Linked Immunosorbent Assay (ELISA)

Cecal samples were collected to detect the concentrations of inflammatory factors, including tumor necrosis factor-α (TNF-α), interleukin-10 (IL-10), interleukin-22 (IL-22) and interferon-γ (IFN-γ), using a competitive enzyme-linked immunosorbent assay (Beyotime, Shanghai, China). All assays were conducted following the protocols provided by the manufacturer.

### 2.9. Measurements of Antioxidant Activity and Lipid Peroxidation

Portions of the cecal segments were homogenized, and clarified lysates were acquired by centrifugation at 200 g for 10 min at 4 °C. The resulting tissue extracts were stored at −80 °C for subsequent antioxidant activity analysis. The activities of superoxide dismutase (SOD), catalase (CAT), and glutathione peroxidase (GPx), as well as the total antioxidant capacity (T-AOC) and malondialdehyde (MDA) content, were assayed using five commercial kits (Beyotime, China) and colorimetric methods. All experimental procedures followed the manufacturer’s instructions.

### 2.10. Microbial Sequencing

Mouse fecal samples were collected and preserved at −80 °C for storage and subsequent analysis. Microbial sequencing followed established protocols [22]. Initially, genomic DNA from bacteria was extracted and amplified using barcoded primers that target the V3–V4 region of the 16S rRNA gene. The PCR products were subsequently subjected to high-throughput pyrosequencing on the Illumina MiSeq platform. Operational taxonomic units (OTUs) were defined by clustering the reads with ≥97% sequence identity. Linear discriminant analysis effect size (LefSe) was employed to assess and quantify biomarkers specific to each experimental group.

### 2.11. Western Blotting

Total protein from cecal segments was extracted, and 20 µg of protein was separated using 10% sodium dodecyl sulfate–polyacrylamide gel electrophoresis (SDS-PAGE). The proteins were then transferred onto membranes and blocked with 5% skim milk for 2 h at room temperature. The membranes were incubated overnight at 4 °C with rabbit anti-mouse primary antibodies, including beta-actin (β-actin, 1:2000; Abcam, ab8227), Claudin-1 (1:1000; Abcam, ab211737), epithelial cadherin (E-cadherin, 1:1000; Abcam, ab314063), zonula occludens-1 (ZO-1, 1:1000; Proteintech, San Diego, CA, USA, 21773-1-AP), kelch-like ECH-associated protein 1 (Keap1, 1:1000; CUSABIO, CSB-PA012147LA01HU), phosphorylated nuclear factor erythroid 2-related factor 2 (p-Nrf2, 1:1000; CUSABIO, Wuhan, China, CSB-PA190654) and inducible nitric oxide synthase (iNOS, 1:1000; Cell signaling, Danvers, MA, USA, 13120S). After washing with tris-buffered saline with tween (TBST), the membranes were incubated with goat anti-rabbit IgG (1:5000, Beyotime, China) for 2 h at 37 °C. The protein bands’ gray values were quantified using ImageJ 4.0.2 software (Bethesda, MD, USA).

### 2.12. Statistical Analysis

The data are presented as the mean ± SD. Differences between groups were analyzed using independent-samples *t*-tests or analysis of variance (ANOVA) followed by Fisher’s least significant difference (LSD) post hoc tests. A *p* value of 0.05 or less was considered statistically significant. Differences were denoted as follows: * *p* ≤ 0.05; ** *p* ≤ 0.01; *** *p* ≤ 0.001; and ns, not significant.

## 3. Results

### 3.1. Esculetin Exhibits Broad-Spectrum Antibacterial Activity

**Figure 2:**
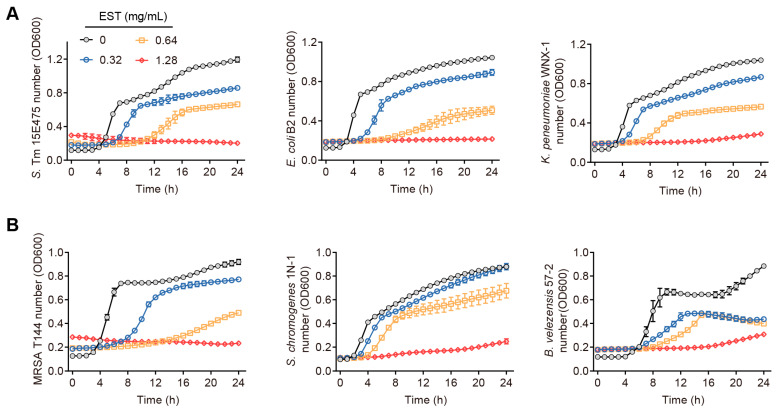
Antibacterial activities of EST on diverse pathogenic bacteria. (**A**) Growth curves of *S*. Tm, *E. coli*, and *K. pneumonia* with EST supplementation. (**B**) Growth curves of MRSA, *S. chromogenes*, and *B. velezensis* with EST supplementation.

Our findings demonstrate that coumarin, EST, fraxetin, daphnetin, scopoletin and 4-methyldaphnetin possess broad-spectrum antibacterial effects against a range of MDR bacteria (Table S1). Interestingly, these six coumarins demonstrated greater antimicrobial activity against Gram-negative bacteria than against Gram-positive bacteria. To further investigate the potential of coumarins in treating MDR *S*. Tm-mediated infectious diseases, we selected EST for detailed mechanistic studies due to its superior antioxidant activity and well-established safety profile among these compounds [23,24]. To evaluate the antibacterial activity of EST, we measured the growth curves of various MDR bacteria by co-culturing EST with different Gram-negative and Gram-positive bacteria, including *S*. Tm 15E475 (*mcr*-3), *E. coli* B2 (*mcr*-1), carbapenem-resistant *K. pneumonia* WNX-1, methicillin-resistant *Staphylococcus aureus* (MRSA) T144, *S. chromogenes* 1N-1, and *B. velezensis* 57-2, respectively, for 24 h. The results demonstrated that EST effectively inhibited the growth of these pathogenic bacteria in a dose-dependent manner (Figure 2A,B), indicating that EST possesses robust antibacterial activity against MDR pathogens. This effect was particularly pronounced in the *S*. Tm 15E475 isolate, which is resistant to colistin.

### 3.2. Esculetin Inhibits the Virulence and Cell Invasion of MDR S. Tm

**Figure 3:**
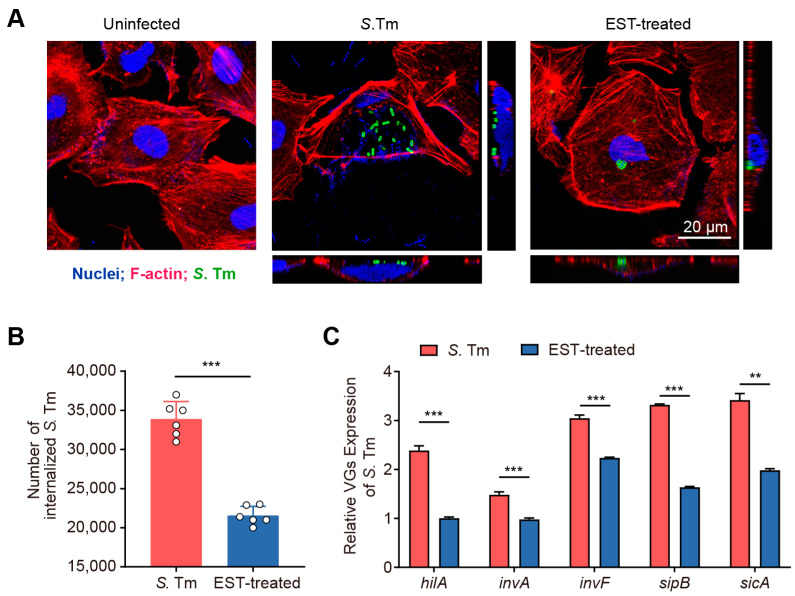
EST reduces the invasion of *S*. Tm into intestinal epithelial cells. (**A**) Confocal images showing internalized *S*. Tm 15E475 (green) in IEC-6 cells. The 3D images were acquired using confocal laser scanning microscopy (CLSM), where F-actin was visualized with rhodamine phalloidin (red) and nuclei with DAPI (blue). (**B**) The number of internalized *S*. Tm 15E475 in IEC-6 cells with or without EST treatment. (**C**) Relative expression of virulence genes (VGs) of *S*. Tm 15E475 with or without EST treatment. Results represent the mean ± standard error. *p* values were calculated using independent-samples *t*-tests. ** *p* ≤ 0.01; *** *p* ≤ 0.001.

The intestinal epithelium functions as a vital physical barrier, protecting against external stressors and pathogens [25]. *S*. Tm utilizes T3SS-1 to secrete effector proteins that facilitate the rearrangement of the actin cytoskeleton, enabling the invasion of IECs [6]. To investigate the effect of EST on the invasive capacity of *S*. Tm into IECs, we performed an invasion assay using the IEC-6 cell line. Our results demonstrated that *S*. Tm actively invaded IEC-6 cells and caused significant morphological damage (Figure 3A). Conversely, EST treatment protected the cells from *S*. Tm-induced impairment, reducing the number of internalized *S*. Tm by 57.23% (*p* < 0.001), compared to cells without EST supplementation (Figure 3B). Correspondingly, the expression of virulence genes (VGs) associated with T3SS-1 of *S*. Tm in the EST-treated group was 1.4- to 2.4-fold lower than in the *S*. Tm-infected group (Figure 3C). Among these virulence genes, *hilA*, a key regulator of SPI-1, directly activates downstream genes such as *inv*, *sic*, *sip*, and other effector genes [26]. Thus, EST may suppress the *hilA*-*inv*-*sic*/*sip* pathway, thereby reducing the expression of T3SS-1 virulence factors in *S*. Tm and preventing its invasion of IECs. Collectively, our findings indicate that EST significantly restricts the growth and invasion of *S*. Tm into intestinal epithelial cells.

### 3.3. Esculetin Decreases S. Tm Load and Disease Severity in Infected Mice

**Figure 4:**
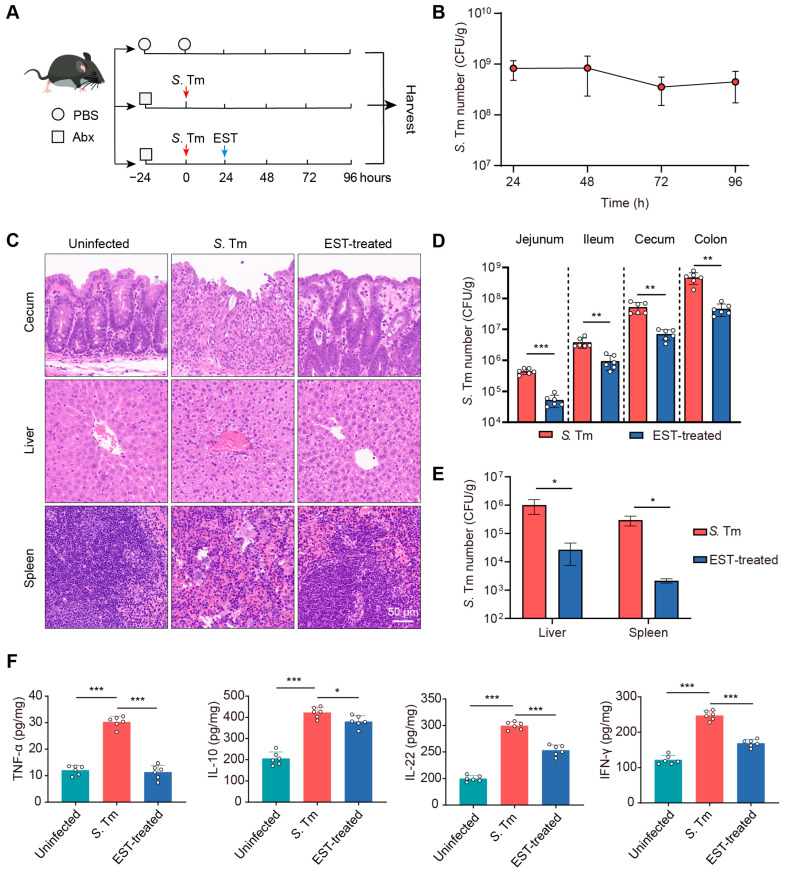
EST decreases *S*. Tm loads and disease severity in infected mice. (**A**) Experimental design for the establishment of *S*. Tm-infected and EST-treated mouse models. (**B**) *S*. Tm shedding in feces of mice for 96 h. (**C**) Representative H&E staining images of the cecum, liver, and spleen in uninfected, *S*. Tm-infected, and EST-treated mice. (**D**) *S*. Tm loads in the jejunum, ileum, cecum, and colon. (**E**) *S*. Tm loads in the liver and spleen. (**F**) Inflammatory cytokine levels in the cecum. Results represent the mean ± standard error. *p* values were calculated using independent-samples *t*-tests (**D**,**E**) or one-way ANOVA followed by Fisher’s LSD post hoc tests (**F**). * *p* ≤ 0.05; ** *p* ≤ 0.01; *** *p* ≤ 0.001.

To assess the therapeutic potential of EST against MDR *S*. Tm-mediated enteric infection, we established *S*. Tm-infected and EST-treated mouse models (Figure 4A). In the EST-treated group, mice received a single administration of EST (100 mg/kg) following MDR *S*. Tm infection. The successful establishment of the *S*. Tm infection model was confirmed by consistent *S*. Tm shedding in the feces for 96 h (Figure 4B). Subsequently, a histological analysis of cecum, liver, and spleen from the various groups was conducted using H&E staining. In *S*. Tm-infected mice, we observed cecal wall shedding and inflammatory infiltration. In contrast, EST treatment significantly ameliorated these conditions, enhancing intestinal epithelial barrier integrity and reducing inflammatory infiltration (Figure 4C). Furthermore, EST-treated mice exhibited reduced disease severity, evidenced by decreased neutrophil infiltration, hemorrhage, cell degeneration, and cell necrosis in the liver and spleen compared to the *S*. Tm-infected mice (Figure 4C). To elucidate the mechanism by which EST alleviates *S*. Tm-induced disease, we quantified the *S*. Tm loads in various intestinal segments and abdominal organs (Figure 4D,E). *S*. Tm primarily colonized in the cecum and colon, and EST treatment significantly decreased *S*. Tm loads by 3- to 10-fold in diverse intestinal segments (Figure 4D). Additionally, EST administration markedly reduced *S*. Tm loads in the liver and spleen by over 35-fold (Figure 4E), indicating the effective inhibition of MDR *S*. Tm proliferation and expansion.

Inflammation can foster the growth of *S*. Tm and promote its transmission in the intestinal lumen [27]. Therefore, we measured the inflammatory cytokine levels in the cecum of mice (Figure 4F). In the *S*. Tm group, infection caused significant increases in TNF-α, IL-10, IL-22, and IFN-γ levels by 150.6%, 105.4%, 49.8%, and 103.4%, respectively, compared to the uninfected controls. In contrast, EST treatment significantly attenuated these inflammatory responses, decreasing cytokine levels by 11.9–166.1% (*p* = 0.000–0.019), compared to the *S*. Tm group. Collectively, EST markedly reduced *S*. Tm loads and the severity of *S*. Tm-induced diseases, demonstrating robust therapeutic potential against MDR bacterial infections in vivo.

### 3.4. Esculetin Alleviates the S. Tm-Induced Microbial Dysbiosis

**Figure 5:**
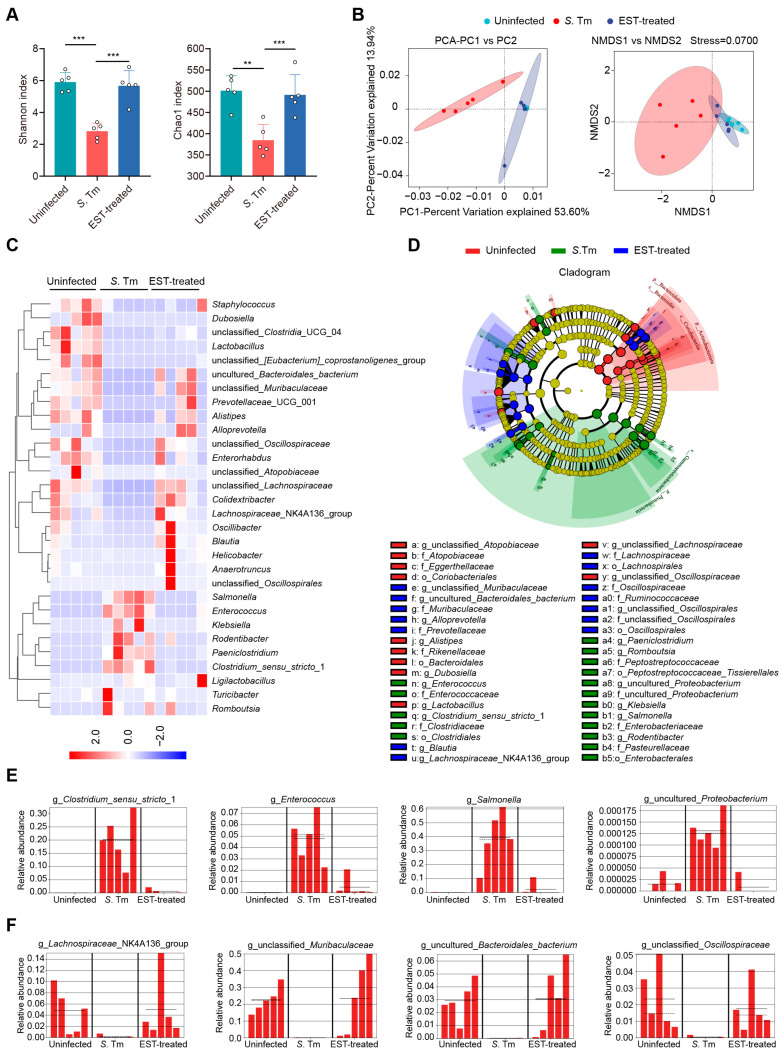
EST improves the microbial barrier of *S*. Tm-infected mice. (**A**,**B**) α-diversity and β-diversity analyses. (**A**) α-diversity analysis (Shannon and Chao1 indexes) of cecal microbiota richness and diversity. (**B**) Principal component analysis (PCA) and nonmetric multidimensional scaling (NMDS) score plot based on the binary Jaccard distance metrics, with each symbol representing one sample. (**C**) Heatmap analysis illustrating changes in relative abundance of the top 30 bacterial species of the cecal microbiota. (**D**) The cladogram displays taxonomic tree of differentially abundant taxa. (**E**,**F**) Relative abundance of key genera in the cecal microbiota based on the LefSe results. Solid and dashed lines indicate the mean and median, respectively. Results represent the mean ± standard error. *p*-values were calculated using the independent-samples *t*-test. ** *p* ≤ 0.01; *** *p* ≤ 0.001.

The gut microbiota plays a crucial role in providing resistance to pathogen invasion and infection, referred to as colonization resistance [28]. Hence, we collected cecal contents from mice to decipher the influence of EST on gut microbiota composition in *S*. Tm-infected mice. Of note, α-diversity analysis (Shannon and Chao1 indexes) revealed a significant decrease in the richness and diversity of cecal microbiota in *S*. Tm-infected mice compared to uninfected mice. However, EST treatment reversed these indexes, restoring richness and diversity (Figure 5A). β-diversity analysis further demonstrated a distinct separation of gut microbiota composition in EST-treated mice compared to *S*. Tm-infected mice (Figure 5B), indicating that EST significantly altered the composition of the gut microbiota in *S*. Tm-infected mice. Specifically, heatmap analysis revealed that *S*. Tm infection increased the relative abundance of pathogenic bacteria such as *Salmonella*, *Enterococcus*, *Klebsiella*, and *Rodentibacter* spp., respectively. Strikingly, EST treatment suppressed these pathogenic bacteria and restored the richness of the top 30 bacteria to levels similar to those uninfected mice (Figure 5C). To identify the specific bacterial taxa associated with *S*. Tm infection and EST treatment, we performed a linear discriminant analysis (LDA) effect size (LEfSe) analysis. The cladogram showed the predominant bacteria and the greatest differences in taxa among the three communities (Figure 5D). Predominant bacteria in the cecal microbiota of *S*. Tm-infected mice included *Clostridium*_*sensu*_*stricto*_1, *Enterococcus*, *Salmonella*, and uncultured_*Proteobacterium* (Figure 5E). In contrast, EST treatment increased the relative abundance of beneficial bacteria such as *Lachnospiraceae*_NK4A136_group, unclassified_*Muribaculaceae*, uncultured_*Bacteroidales*_*bacterium*, and unclassified_*Oscillospiraceae* (Figure 5F), acknowledged as ecological gatekeepers in the healthy gut microbiota [29,30]. Altogether, the upregulation of microbial diversity and beneficial microbes suggests that EST improves the microbial barrier in *S*. Tm-infected mice, thereby aiding in the combat against *S*. Tm infection in the gut.

### 3.5. Esculetin Enhances the Physical Intestinal Barrier against S. Tm Infection

**Figure 6:**
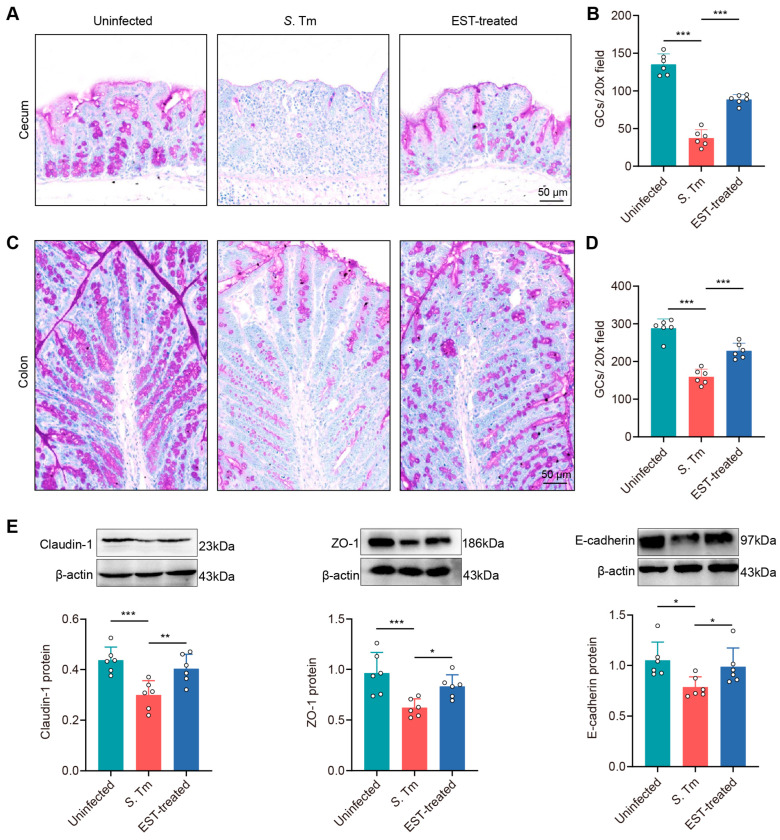
EST enhances the physical intestinal barrier against *S*. Tm infection. (**A**–**D**) Images of PAS-stained sections and the quantification of goblet cells in the cecum (**A**,**B**) and colon (**C**,**D**). (**E**) Expression of Claudin-1, ZO-1, and E-cadherin protein in the cecum. Protein levels were normalized relative to β-actin (*n* = 6). Results represent the mean ± standard error. *p* values were calculated using one-way ANOVA followed by Fisher’s LSD post hoc test. * *p* ≤ 0.05; ** *p* ≤ 0.01; *** *p* ≤ 0.001.

To investigate whether EST treatment augments intestinal mucosal integrity in *S*. Tm-infected mice, we examined changes in goblet cell count and the expression of tight junction proteins in mucosal epithelium. PAS staining images showed a pronounced decrease in goblet cell numbers in the cecum and colon of *S*. Tm-infected mice compared with uninfected mice by 262.1% (*p* < 0.001) and 80.9% (*p* < 0.001), respectively. In contrast, EST supplementation significantly elevated these values (Figure 6A–D).

In addition, the Western blot assay showed a decrease in the expression of three tight junction proteins by 46.1% (Claudin-1, *p* = 0.001), 54.9% (ZO-1, *p* = 0.001) and 33.8% (E-cadherin, *p* = 0.012), respectively, in the cecal epithelium of *S*. Tm-infected mice compared to uninfected mice (Figure 6E). Conversely, EST treatment attenuated this impairment, with an increased expression of Claudin-1, ZO-1, and E-cadherin by 25.5–34.7% (*p* = 0.006–0.047) in EST-treated mice, compared to *S*. Tm-infected mice. Altogether, these findings indicate that EST effectively enhances the physical intestinal barrier against *S*. Tm infection.

### 3.6. Esculetin Ameliorates S. Tm-Induced Intestinal Oxidative Stress via Activating the Nrf2 Pathway

**Figure 7:**
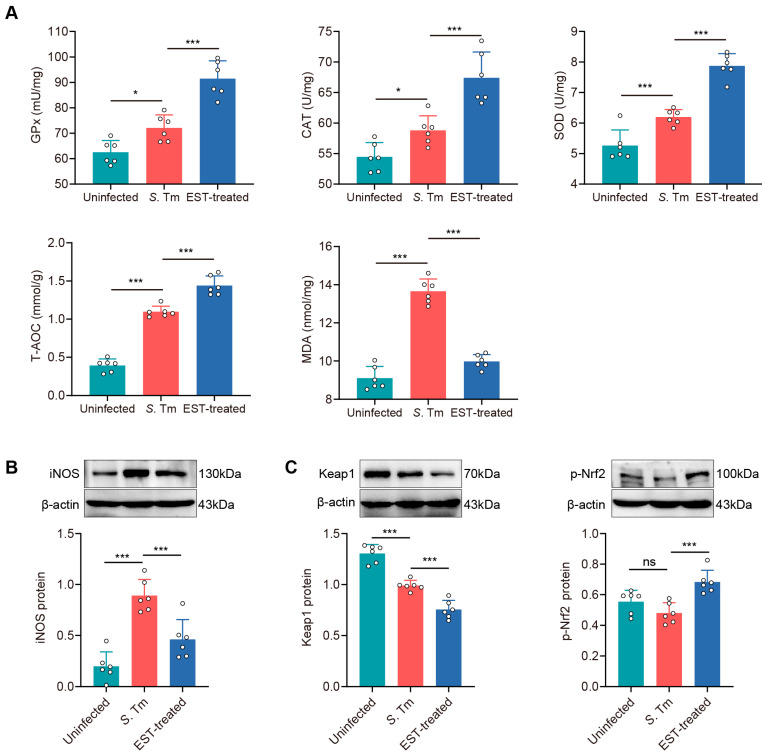
EST ameliorates *S*. Tm-induced intestinal oxidative injury via activating the Nrf2 pathway. (**A**) Concentrations of antioxidant enzymes (GPx, CAT and SOD), T-AOC and MDA in the cecum (*n* = 6). (**B**,**C**) Cecal iNOS (**B**), Keap1 and p-Nrf2 (**C**) protein expression in uninfected, *S*. Tm and EST-treated groups. Relative protein levels were normalized to β-actin (*n* = 6). Results represent the mean ± standard error of the mean. *p*-values were calculated using one-way ANOVA followed by Fisher’s LSD post hoc tests. * *p* ≤ 0.05; *** *p* ≤ 0.001; ns, not significant.

To assess whether *S*. Tm infection induces oxidative stress in the gut, we examined five antioxidant parameters, including antioxidant enzymes (GPx, SOD, and CAT), total antioxidant capability (T-AOC), and malondialdehyde (MDA), in the cecum (Figure 7A). Antioxidant enzymes and T-AOC levels were significantly enhanced by 15.3% (GPx, *p* = 0.011), 8.0% (CAT, *p* = 0.034), 17.7% (SOD, *p* = 0.001), and 177.8% (T-AOC, *p* < 0.001) in *S*. Tm-infected mice compared to uninfected mice. Conversely, MDA levels, indicative of lipid peroxidation and endogenous oxidative damage, were elevated by 49.9% (*p* < 0.001) in the cecum of *S*. Tm-infected mice compared to uninfected mice. Interestingly, EST treatment further increased antioxidant enzymes while reducing MDA levels (Figure 7A). These results indicated that EST treatment significantly improves the antioxidant capacity of *S*. Tm-infected mice. The activation of the Nrf2 pathway is known to play a crucial role in attenuating oxidative damage in various diseases [31]. We observed that EST treatment effectively reversed the increase in iNOS protein levels by *S*. Tm infection (Figure 7B). Meanwhile, EST treatment reduced Keap1 protein levels by 31.1% (*p* < 0.001) and significantly enhanced p-Nrf2 protein levels by 42.4% (*p* < 0.001) compared with the *S*. Tm group. These results indicate that EST activates the Nrf2 pathway by enhancing Nrf2 phosphorylation and reducing Keap1 expression, thereby ameliorating *S*. Tm-induced intestinal oxidative injury.

## 4. Discussion

The emergence and rapid spread of MDR *S*. Tm poses a grave threat to global public health [32], underscoring the urgent need for alternative antimicrobial approaches. While natural products derived from dietary and medicinal plants have long been utilized to treat a variety of diseases [33,34], their potential against MDR bacteria remains relatively unexplored. Our investigation revealed that many simple coumarins have broad-spectrum antibacterial activity against MDR bacteria, such as colistin-resistant Enterobacteriaceae and methicillin-resistant *Staphylococcus aureus*. Given the frequent exposure of human diets to coumarin derivatives [13], we evaluated the therapeutic potential of EST, a representative simple coumarin, against MDR *S*. Tm-induced enteric infection. Remarkably, EST inhibited the virulence associated with T3SS-1 of *S*. Tm, thereby impeding its invasion of intestinal epithelial cells. Furthermore, EST significantly reduced *S*. Tm loads in various intestinal segments and organs and alleviated intestinal barrier dysfunction. These findings highlight natural coumarins as promising antibacterial agents to restrict MDR foodborne pathogens.

*S*. Tm employs its T3SS-1 virulence factors to invade the intestinal epithelium, triggering inflammatory responses [26]. Inflammation, in turn, elicits oxidative stress, facilitating the expansion of *S*. Tm in the gut [6]. Inflammatory cytokines can stimulate the release of reactive nitrogen species (RNS) and reactive oxygen species (ROS), thereby producing tetrathionate and nitrate, which serve as terminal electron acceptors for *S*. Tm respiration, providing it with a growth advantage [6]. Therefore, targeting T3SS-1 and consequent intestinal inflammation holds promise as a therapeutic strategy against *S*. Tm infection and associated diseases.

Fortunately, we found that EST inhibited a series of T3SS-1 virulence genes and decreased the internalization of *S*. Tm, thus mitigating *S*. Tm-induced cellular damage. Similar inhibitors targeting T3SS derived from natural products have been reported. For instance, myricetin, a flavonoid compound, exerts a strong inhibitory effect on the T3SS-mediated virulence in *S*. Tm [35]. Besides *S*. Tm, other pathogens employing the T3SS include enterohemorrhagic and enteropathogenic *E. coli* (EHEC and EPEC), *Chlamydia* species, *Shigella* spp., *Pseudomonas* spp., *Vibrio* spp., and *Yersinia pestis* [36]. Therefore, the exploration of effective T3SS inhibitors represents a powerful strategy against antibiotic resistance in these multidrug-resistant (MDR) pathogens, with dietary coumarins potentially serving as a valuable screening library. Additionally, previous reports have demonstrated that coumarin derivatives possess antimicrobial properties against *Salmonella* by inhibiting biofilm formation, competing for iron, or targeting DNA gyrase [37,38]. Nevertheless, few studies have investigated the influence of coumarins on intestinal barrier function in *S*. Tm-infected mice and the associated therapeutic mechanisms.

Here, we demonstrated that EST treatment significantly decreased the levels of inflammatory factors (TNF-α, IL-10, IL-22, and IFN-γ) in the cecum of *S*. Tm-infected mice. This is consistent with previous studies that report that EST has anti-inflammatory effects in various diseases such as early sepsis [39], septic cardiomyopathy [40], and ulcerative colitis [41]. During infection, IFN-γ can boost the expression of iNOS to produce NO and ROS, thereby promoting *S*. Tm growth [42,43]. Consistent with the reduced levels of IFN-γ, we observed that EST treatment inhibited the protein expression of iNOS in the cecum of *S*. Tm-infected mice as well. Meanwhile, EST treatment significantly reduced the protein expression of Keap1, and enhanced the protein expression of p-Nrf2 in the cecum, compared with the *S*. Tm group. Under normal conditions, Nrf2 in the cytoplasm remains inactive due to its binding to the inhibitor Keap1. When activated, p-Nrf2 separates from Keap1 and translocates to the nucleus, where it directly binds to antioxidant response elements (AREs) in the promoters of target genes, enhancing the production of antioxidant enzymes [31]. Consequently, our findings suggest that EST mitigates intestinal inflammation and oxidative damage by activating the Nrf2 signaling pathway.

Intestinal inflammation is also closely linked with dysbiosis in the gut microbiota [44]. Our findings demonstrated that the cecal microbiota of *S*. Tm-infected mice is marked by decreased microbial diversity, an expansion of the pathobiont *Enterococcus*, and increased levels of pathogenic *Salmonella*, *Klebsiella*, and *Rodentibacter* spp. Strikingly, EST treatment not only suppressed the abundance of pathogenic bacteria but also significantly enhanced the relative abundance of beneficial bacteria, including *Lachnospiraceae*_NK4A136_group, unclassified_*Muribaculaceae*, uncultured_*Bacteroidales*_*bacterium*, and unclassified_*Oscillospiraceae*. Among these bacteria, *Lachnospiraceae* are known for producing butyrate, and their presence is inversely related to inflammation levels [45]. Butyrate has been demonstrated to decrease the expression of *Salmonella* invasion genes [46], suggesting that EST treatment might inhibit *S*. Tm infection by influencing the microbial composition and metabolites. Therefore, it is important to further explore the therapeutic effects of butyrate-producing bacteria or butyrate supplementation on MDR *S*. Tm infection. Additionally, there are other limitations to our work. Although we demonstrated that EST inhibited the virulence of *S*. Tm, thereby preventing its cellular invasion, the direct evidence linking the reduced virulence expression of *S*. Tm and intestinal inflammation remains unclear. Future studies are crucial to investigate how coumarins or other virulence inhibitors modulate intestinal inflammation and ultimately treat MDR enteric infections.

## 5. Conclusions

In summary, the simple coumarin, EST, effectively downregulates the T3SS-1 virulence expression of MDR *S*. Tm, thereby preventing its invasion into intestinal epithelial cells. Meanwhile, EST suppresses the proliferation and expansion of *S*. Tm and ameliorates intestinal barrier dysfunction and oxidative damage by activating the Nrf2 pathway. These findings underscore the potential of natural coumarins as promising candidates to combat enteric infections associated with MDR pathogenic bacteria.

## Figures and Tables

**Table 1:** Sequences of primers used in this study.

Target	Primers (5′-3′)	Reference
*rpoD*-F	GTGAAATGGGCACTGTTGAACTG	[20]
*rpoD*-R	TTCCAGCAGATAGGTAATGGCTTC	
*hilA*-F	TGTCGGAAGATAAAGAGCAT	
*hilA*-R	AAGGAAGTATCGCCAATGTA	
*invA*-F	GAAATTATCGCCACGTTCGGGCAA	
*invA*-R	TCATCGCACCGTCAAAGGAACC	
*invF*-F	GCAGGATTAGTGGACACGAC	[21]
*invF*-R	TTTACGATCTTGCCAAATAGCG	
*sicA*-F	ATTTGGGATGCCGTTAGTGAAG	
*sicA*-R	TAAACCGTCCATCATATCTTGAGG	
*sipB*-F	GCCGATGAAATTGTGAAGGC	This study

## Data Availability

16S rRNA sequencing data (PRJNA1141740) are available in the National Center for Biotechnology Information database.

## Supplementary Materials

The following supporting information can be downloaded at https://www.mdpi.com/article/10.3390/antiox13101170/s1. Table S1. Antibacterial Activities of Coumarin and Its Simple Derivatives.
