# Supplementary material for: Esculetin Combats Multidrug-Resistant Salmonella Infection and Ameliorates Intestinal Dysfunction via the Nrf2 Pathway
Source: Antioxidants (Basel). 2024 Sep 26;13(10):1170. doi: 10.3390/antiox13101170 (PMC11504508; doi:10.3390/antiox13101170)
Supplement: Supplementary file 1 [file antioxidants-13-01170-s001.zip › antioxidants-3115498-supplementary.pdf]

Table S1. Antibacterial Activities of Coumarin and Its Simple Derivatives.

	Coumarin	Esculetin	Fraxetin	Daphnetin	Scopoletin	4-methyl-daphnetin
Gram-negative bacteria			MIC (mg/mL)			
E. coli ATCC25922	5.12	0.64	0.32	0.32	1.28	1.28
E. coli B2	>5.12	1.28	1.28	5.12	>5.12	5.12
S. Tm 15E475	5.12	1.28	0.64	0.64	5.12	1.28
S. Tm ATCC14028	5.12	0.64	0.32	0.32	1.28	1.28
A. baumannii 17918	0.64	0.64	0.32	0.32	1.28	0.32
K. pneumoniae WNX-1	5.12	1.28	1.28	1.28	5.12	1.28
A. veronii CVCC3700	5.12	0.32	0.08	0.32	5.12	0.08
P. aeruginosa PAO-1	5.12	>5.12	2.56	>5.12	>5.12	5.12
Gram-positive bacteria						
S. aureus ATCC29213	>5.12	1.28	0.64	5.12	>5.12	1.28
S. chromogenes 1N-1	>5.12	1.28	0.16	0.64	0.64	0.64
S. haemolyticus 2N-1	>5.12	1.28	0.64	5.12	>5.12	2.56
B. cereus CAU45	>5.12	2.56	0.64	5.12	5.12	1.28
C. welchii 19SX1RK45	>5.12	5.12	0.64	2.56	>5.12	5.12

MIC, minimum inhibitory concentration; *Escherichia coli, E. coli*; *Salmonella enterica* serovar Typhimurium, *S*. Tm; *Acinetobacter baumannii, A. baumannii*; *Klebsiella pneumoniae, K. pneumoniae*; *Aeromonas vickeri, A. veronii*; *Pseudomonas aeruginosa, P. aeruginosa*; *Staphylococcus aureus, S. aureus*; *Staphylococcus chromogenes, S. chromogenes*; *Staphylococcus haemolyticus, S. haemolyticus*; *Bacillus cereus, B. cereus*; *Clostridium welchii, C. welchii*.
